# Leptin Locally Synthesized in Carotid Atherosclerotic Plaques Could Be Associated With Lesion Instability and Cerebral Emboli

**DOI:** 10.1161/JAHA.112.001727

**Published:** 2012-10-25

**Authors:** Jacob Schneiderman, Katrin Schaefer, Frank D. Kolodgie, Naphtali Savion, Shlomo Kotev-Emeth, Rima Dardik, Amos J. Simon, Moshe Halak, Clara Pariente, Isaac Engelberg, Stavros Konstantinides, Renu Virmani

**Affiliations:** 1 Department of Vascular Surgery and the Gottesdiener Vascular Biology Laboratory, Sheba Medical Center, Sackler Faculty of Medicine, Tel Aviv University, Israel (J.S., M.H., I.E.); 2 Institute of Thrombosis and Hemostasis, Sheba Medical Center, Sackler Faculty of Medicine, Tel Aviv University, Israel (R.D.); 3 Sheba Cancer Research Center and Institute of Hematology, Sheba Medical Center, Sackler Faculty of Medicine, Tel Aviv University, Israel (A.J.S.); 4 Institute of Endocrinology, Sheba Medical Center, Sackler Faculty of Medicine, Tel Aviv University, Israel (C.P.); 5 Department of Cardiology and Pulmonary Medicine, University Medicine Goettingen, Germany (N.S., S.K.-E.); 6 CVPath Institute, Inc, Gaithersburg, MD (K.S.); 7 Goldschleger Eye Research Institute, Sackler Faculty of Medicine, Tel Aviv University, Israel (F.D.K., R.V.); 8 Department of Cardiology, Democritus University of Thrace, Alexandroupolis, Greece (S.K.)

**Keywords:** atherosclerosis, leptin, macrophages, smooth muscle cells, tumor necrosis factor-α

## Abstract

**Background:**

Unstable carotid plaques cause cerebral emboli. Leptin promotes atherosclerosis and vessel wall remodeling. We hypothesized that carotid atherosclerotic lesion instability is associated with local leptin synthesis.

**Methods and Results:**

Carotid endarterectomy plaques from symptomatic (n=40) and asymptomatic patients with progressive stenosis (n=38) were analyzed for local expression of leptin, tumor necrosis factor (TNF)-α, and plasminogen activator inhibitor type 1. All lesions exhibited advanced atherosclerosis inclusive of thick- and thin-cap fibroatheromas or lesion rupture. Symptomatic lesions exhibited more plaque ruptures and macrophage infiltration (*P*=0.001 and *P*=0.05, respectively). Symptomatic plaques showed preferential leptin, TNF-α, and plasminogen activator inhibitor type 1 transcript (*P*=0.03, *P*=0.04, and *P=*0.05, respectively). Leptin mRNA and antigen in macrophages and smooth muscle cells were confirmed by in situ hybridization and immunohistochemistry. Plasma leptin levels were not significantly different between groups (*P*=1.0), whereas TNF-α was significantly increased in symptomatic patients (*P*=0.006). Human aortic smooth muscle cell culture stimulated by TNF-α, lipopolysaccharide, or lipoteichoic acid revealed 6-, 6.7-, and 6-fold increased secreted leptin antigen, respectively, at 72 hours (*P*<0.05).

**Conclusions:**

Neurologically symptomatic patients overexpress leptin mRNA and synthesize leptin protein in carotid plaque macrophages and smooth muscle cells. Local leptin induction, presumably by TNF-α, could exert paracrine or autocrine effects, thereby contributing to the pathogenesis of lesion instability.

**Clinical Trial Registration:**

URL: www.Clinicaltrials.gov. Unique identifier: NCT00449306.

## Introduction

Unstable carotid plaques frequently undergo rupture with intraluminal thrombosis, resulting in downstream cerebral emboli and stroke. Complex mechanisms involved in lesion progression and instability include induction of oxidative stress and the recruitment and activation of inflammatory/immune cells capable of synthesizing proatherogenic cytokines.^1^ Additionally, synthesis of proteolytic enzymes like matrix metalloproteinases^2^ or cathepsins^3^ and angiogenic vascular endothelial growth factor-A, which underlie intraplaque hemorrhage,^4^ can lead to degradation of the fibrous cap and undermine the physical stability of the lesion. Many proatherogenic and proteolytic effects have been attributed at least in part to leptin activity. Thus, in addition to its pleiotropic hormonal functions regulating metabolic, immune, and endocrine functions,^5^ leptin has been implicated in the process of atherosclerosis via multiple avenues. For example, leptin has been shown to stimulate endothelial cells to increase oxidative stress through the generation of reactive oxygen species.^6^ Those enhance the expression of intracellular adhesive molecules^7^ and monocyte chemoattractant protein-1,^8^ both known to be involved in atherogenesis. Reactive oxygen species also exert elastolytic activity on the vessel wall capable of remodeling it. Leptin activates matrix metalloproteinase-2, upregulates matrix metalloproteinase-9, and stimulates endothelial cells to generate pathological angiogenesis via vascular endothelial growth factor-A synthesis,^9^ thus promoting plaque destabilization.^4,10^ Leptin also enhances atherosclerosis by inducing vascular smooth muscle cell (SMC) proliferation and migration through mitogen-activated protein kinase and phosphatidylinositol 3-kinase activation.^11^ SMCs also are stimulated to synthesize matrix metalloproteinase, tissue inhibitor of metalloproteinases,^12^ and angiotensin II receptor type 1.^13^ Human leptin increases cytokine production^14–15^ and stimulates proliferation of human circulating monocytes^16^ and T lymphocytes in peripheral blood.^17^ Leptin stimulates various immune cells involved in atherosclerosis to produce proinflammatory cytokines, which in turn form a feedback loop by promoting leptin synthesis in vivo,^18^ which suggests an autocrine/paracrine mode of action.

The impact of leptin on atherosclerosis and neointimal hyperplasia has been confirmed in a few experimental animal models^19–21^ and numerous clinical studies. Notably, clinical data have revealed that leptin expression is associated with obesity, insulin resistance, and metabolic syndrome.^22^ Moreover, hyperleptinemia is a surrogate biomarker for coronary vascular events,^23^ coronary restenosis after balloon angioplasty,^24^ and hemorrhagic stroke.^25^

Adipocytes currently are considered the sole source of plasma leptin,^26^ with the activity of leptin at the tissue level being mediated through an interaction with the leptin receptor (ObR).^27^ A recent study of carotid atherosclerotic plaques has revealed that ObR is preferentially synthesized in lesions from neurologically symptomatic patients.^28^ Thus, it is reasonable that circulating leptin could be interacting with accumulated ObR in symptomatic carotid plaques. We hypothesized that leptin can be synthesized locally within carotid atherosclerotic lesions and could in part be responsible for the underlying plaque instability that leads to neurological symptoms. In addition, in light of numerous clinical data suggesting a causative linkage between inflammation and enhanced atherosclerosis,^29^ we further hypothesized that tumor necrosis factor (TNF)-α might be involved in leptin induction within the carotid plaque.

## Methods

### Patients

Carotid plaques were collected from 78 patients undergoing endarterectomy for extracranial high-grade internal carotid artery stenosis (>70% luminal narrowing for symptomatic patients and >80% for asymptomatic patients). Forty of the patients were symptomatic, as defined by the North American Symptomatic Carotid Endarterectomy Trial (NASCET) classification,^30^ and their clinical presentation was transient ischemic attacks (n=27) or stroke (n=13) within the 3 months preceding tissue collection. The remaining 38 patients were clinically asymptomatic, exhibiting progressive ipsilateral internal carotid artery stenosis exceeding 80% on serial Doppler examinations. This study was approved by the local ethics committee, and written informed consent was obtained from all patients.

### Clinical Data

Clinical data (summarized in Table 1) were collected for all participating subjects. Hypertension was established by history, medical therapy, and blood pressure measurements of >130/85 mm Hg. Confirmation of diabetic status (types I and II) was based on history, medications, blood glucose, and hemaglobin A1c (>6.5%). The degree of internal carotid artery stenosis was established by 2 consecutive Doppler examinations before surgery. Computed tomography angiography was selectively performed in 65% of the patients (n=51) to surmise the degree of luminal compromise and to resolve controversial Doppler results. A senior neurologist evaluated all patients before and after surgery. Patients with medical history or clinical symptoms of stroke underwent a brain computed tomography scan and were operated on within 8 days to 2 months after the cerebrovascular event. Symptomatic patients presenting transient ischemic attacks underwent surgery within 2 days to 3 months after the episode.

**Table 1.** Demographics, Cardiovascular Risk Factors, and Medications

	Symptomatic	Asymptomatic	*P*
Number	40	38	NS
Age, y, range	37–82	54–80	
Age, y, mean±SD	65.0±9.3	68.4±7.5	0.07
Sex, male/female, n	27/13	28/10	0.54
BMI, kg/m^2^, mean±SD	27.8±4.1	28.1±4.3	0.74
HTN, n	31	32	0.45
Diabetes mellitus, n	14	17	0.37
IHD, n	16	21	0.17
Dyslipidemia, n	33	30	0.69
Smoking, n	18	14	0.46
Thrombophilia, n	3	0	0.07
PAD, n	12	12	0.88
ACE inhibitors, n	12	14	0.52
ASA or clopidogrel	36 or 4	37 or 1	0.18
ASA and clopidogrel	14	0	0.001
Statins, n	35	31	0.47

BMI indicates body mass index; HTN, arterial hypertension; IHD, ischemic heart disease; PAD, peripheral arterial disease; ACE inhibitors, angiotensin-converting enzyme inhibitors; and ASA, acetyl salycilic acid.

### Tissue Collection

All plaques were assessed macroscopically during surgery for signs of luminal ulceration, intraplaque hemorrhage, and the presence of a soft or ruptured necrotic core. The segments were ≍8 to 10 mm in length, extending in situ from the common carotid bifurcation into the internal carotid artery. Areas exhibiting the greatest plaque area or macroscopic advanced features of atherosclerosis were selected for analysis. After a brief rinse in normal saline, a 3- to 5-mm segment from the presenting lesion was immediately snap-frozen in liquid nitrogen and set aside at −70°C for RNA analysis. The adjacent plaque was then immersion-fixed in 4% formalin for 8 to 12 hours and subsequently processed for paraffin embedding.

### Immunohistochemistry

Paraffin-embedded carotid plaques were serially sectioned at 4 μm and exposed to antibodies specific for smooth muscle α-actin (dilution 1:400, Dako, Glostrup, Denmark), macrophage marker CD68 (dilution 1:400, Kp-1 clone, Dako), TNF-α (dilution 1:800, Abcam, Cambridge, MA, USA), and TNF-α receptor-1 (dilution, 1:40, Santa Cruz Biotechnology, Santa Cruz, CA, USA). For identification of leptin and its specific receptor (ObR), histological sections were incubated overnight at 4°C with a rabbit polyclonal antibody against human leptin (dilution, 1:200, Santa Cruz Biotechnology) or human leptin receptor (dilution 1:100, Santa Cruz Biotechnology). When necessary, antigen retrieval was performed by steam heat with EDTA (pH 8.0) or citrate (pH 6.0) buffer. Antibody binding was visualized by a polymer-based horseradish peroxidase substrate (EnVision, Dako), with diaminobenzidine as the chromogen and Gill's hematoxylin as counterstain. Sections of normal gastric fundus mucosa and replacement of the primary antibodies by phosphate-buffered saline served as positive and negative controls for leptin-associated antibodies, respectively.

For colocalization of leptin and ObR in SMCs and macrophages, antileptin antibodies were applied as described above, followed by a biotinylated goat anti-rabbit secondary antibody. Sections were stained with the use of a steptavidin-alkaline phosphatase detection system (Vectastain ABC system, Burlingame, CA, USA), and antibody staining was visualized with the Vucan Fast Red chromogen substrate (Biocare Medical, Concord, CA, USA). Subsequently, antibodies against either SMC (1:80) or CD68 (1:200) were applied overnight at 4°C after a sequential application of avidin/biotin (Vector Labs, Burlingame, CA, USA) and protein blocks (Dako), and then sections were labeled and developed as described above.

### Lesion Analysis

The lesions were classified on hematoxylin and eosin– and Movat's pentachrome–stained sections according to the American Heart Association (AHA) criteria, as defined by Stary and modified by Virmani.^31–32^ The presence of macrophage infiltrates and intraplaque vasa vasorum also was assessed by routine light microscopy, performed by 2 investigators in a blinded experiment.

### In Situ Hybridization

Leptin cDNA was generated from human adipose tissue.^33^ Purified complementary DNA was cloned into the pCR II-TOPO vector (Invitrogen, Carlsbad, CA, USA), linearized with *BamHI* and *ApaI* to generate sense and antisense strands, respectively, and verified for correct insertion and sequence (Eurofins MWG Operon, Ebersberg, Germany). Riboprobes were generated by in vitro transcription and labeled by digoxigenin 11-UTP incorporation (Roche, Basel, Switzerland) by using either SP6 (antisense) or T7 (sense) RNA polymerase. The labeled probes were digested with deoxyribonuclease, extracted with phenol/chloroform, and ethanol precipitated. Leptin mRNA expression in situ was examined with a protocol modified from Breitschopf et al.^34^ Briefly, 5-μm-thick paraffin sections were digested with proteinase K (0.005%; 30 minutes at 37°C), followed by overnight incubation with freshly labeled probes at 55°C. Positive signals were visualized by incubation with antidigoxigenin AP (dilution, 1:250 for 2 hours at room temperature; Roche) and nitroblue tetrazolium/5-bromo-4-chloro-3-indolyl-phosphatase (Dako) until color development and were counterstained with Nuclear Fast Red. Human adipose tissue served as positive (labeled antisense) and negative (labeled sense probes) controls.

### RNA Extraction

Total RNA was isolated with the Trizol reagent (Invitrogen) and quantified with a spectrophotometer. First-strand complementary DNA (cDNA) was synthesized by using random hexamers with Superscript II (Gibco BRL, Grand Island, NY, USA). The cDNA prepared from the total RNA was used for quantitative real-time polymerase chain reaction (PCR) analysis for leptin, TNF-α, and plasminogen activator inhibitor type 1 (PAI-1).

### Quantitative Real-Time PCR

Reactions were performed on cDNA with an ABI PRISM 7900HT Sequence Detection System (Applied Biosystems, Carlsbad, CA, USA) and Universal PCR Master Mix (Applied Biosystems) according to the manufacturer's instructions. The TaqMan probes and primers for leptin (assay ID number HS00174877), TNF-α (assay ID number HS00174128), and PAI-1 (assay ID number Hs00167155) were assay-on-demand gene expression products (Applied Biosystems). Total RNA from normal donor carotid arteries (n=3) was used to establish a baseline mRNA level for each gene, defined as 1. All samples from symptomatic and asymptomatic patients were normalized by using the endogenous control gene *Abl (Abelson)* with the fluorescent probe 5′-Fam-CTGGCCCAACGATGGCGA-BHQ-3′.^35^ The primers used were: forward, 5′-GGAGATAACACTCTAAGCATAACTAAAGG-3′, and reverse, 5′-GATGTAGTTGCTTGGGACCCA-3′. Gene expression results for leptin and TNF-α in the plaques were expressed in arbitrary units. Results for leptin mRNA in SMC lysates were presented as differences of normalized Ct values compared to control samples (ΔΔCt). Data were analyzed in SDS 2.3 (Applied Biosystems) and Excel (Microsoft Corp, Redmond, WA, USA) software.

### Determination of Leptin and TNF-α Antigen in the Plasma

Plasma samples were collected from all patients within 2 hours before surgery and stored at −70°C. Circulating levels of leptin were determined with radioimmunoassay kits (Human Leptin RIA kit, Linco Research, Inc, St. Charles, MO, USA). The sensitivity of the leptin assay was 0.5 μg/L, and the interassay coefficient of variation was 3% to 6%. TNF-α plasma levels were assayed with solid-phase enzyme-linked immunosorbent assay (ELISA; R&D, Minneapolis, MN, USA).

### In Vitro Studies in Human Aortic and Human Saphenous Vein SMCs

Normal human SMCs from aorta were purchased from PromoCell (Heidelberg, Germany) and were grown in Dulbecco's modified Eagle medium supplemented with 10% fetal calf serum. Fibroblast growth factor-2 (3 ng/mL) was added every other day until 80% confluence. The assays were performed on confluent cultures (6 to 9 days old) in 35-mm tissue culture dishes (NUNC, Roskilde, Denmark) that were preincubated in fresh medium (1 mL Dulbecco's modified Eagle medium containing 1% fetal calf serum) for 24 hours. Human venous SMCs were isolated by explantation from saphenous veins obtained for cardiac bypass surgery. Cells were cultured in Dulbecco's modified Eagle medium containing 10% fetal calf serum and were used between passages 3 and 6. When cells reached 90% confluence, they were preincubated in Dulbecco's modified Eagle medium containing 1% fetal calf serum for 24 hours.

SMC cultures then were challenged with TNF-α (50 ng/mL, Sigma-Aldrich, St. Louis, MO, USA), lipopolysaccharide (LPS; 100 ng/mL, Sigma-Aldrich), or lipoteichoic acid (LTA; 10 μg/mL, Sigma-Aldrich) for 24, 48, and 72 hours, respectively, and nonstimulated aortic SMCs served as controls. Conditioned media were collected and assayed for leptin antigen level using a Human Leptin ELISA kit (Ray Biotech, Inc, Norcross, GA, USA) according to manufacturer's instructions, where the minimum detectable dose of leptin is typically <6 pg/mL. Cell lysates from human aortic SMC cultures were used to isolate total RNA with an RNeasy kit (Qiagen, Hilden, Germany) and were reverse-transcripted by Superscript II (Invitrogen).

Quantitative PCR was performed for leptin mRNA in a MyiQ Single-Color Real-Time PCR system with SYBR Green I (Bio-Rad, Hercules, CA, USA). Leptin mRNA levels were normalized to 18S. The primer sequences were: leptin, A^2074^ AA GAG GTT TGG GGT CT, and C^2134^ CC ACT GTG TGA CAA AAA C; 18S, 5′-ATGGCCGTTCTTAGTTGGTG-3′ and 5′-GAACGCCACTTGTCCCTCTA-3′.

In vitro leptin antigen studies were performed on 2 commercially available batches of human aortic SMC primary cultures and 3 primary saphenous vein SMC cultures from different individuals.

### Statistical Analysis

Differences in categorical variables (Tables 1 and 2) were determined by the Fisher exact test. mRNA levels between lesions from symptomatic and asymptomatic patients were analyzed by nonparametric methods. Plasma levels of leptin and TNF-α were compared between the groups using a 2-sided Wilcoxon sum test. Relationships between transcript levels for leptin and TNF-α and plasma leptin levels to body mass index were performed by Pearson's correlation. Fold increase of leptin antigen level in cultured SMCs challenged by TNF-α, LPS, and LTA at 3 different time points were assessed by repeated-measures analysis by ANOVA methods. Statistical analysis was performed in SAS 9.1 software (SAS Institute, Inc, Cary, NC), and a value of *P*<0.05 was considered statistically significant.

**Table 2.** Histological Features of the Carotid Plaques

	Symptomatic	Asymptomatic	*P*
Number	40	38	—
AHA class			
III	—	2	—
IV	9	4	0.15
V to VI	31	32	0.45
Plaque rupture, n	16	3	0.001
Necrotic core (>25%)	35	28	0.12
Intraplaque hemorrhage, n	23	18	0.37
Intraluminal thrombosis, n	5	0	0.02
Fibrosis, n	38	36	0.95
Angiogenesis, n	33	27	0.23
Calcification, n	31	32	0.45
Macrophage infiltration, n	39	32	0.05

AHA indicates American Heart Association.

## Results

The purpose of this study was to investigate leptin synthesis in carotid atherosclerotic plaques in association with clinical neurological symptoms. Clinical criteria with regard to demographics, cardiovascular risk factors, and medications (Table 1) were similar in both groups, except for a greater number of patients receiving clopidogrel (Plavix) preoperatively in the symptomatic group (*P*=0.001).

Carotid lesions from both groups were assessed histologically. In both groups, lesions showed features of advanced atherosclerosis, with a similar prevalence of fibroatheromas, with or without a thick cap and calcification (Table 2). Symptomatic patients, however, exhibited a higher incidences of plaque rupture (*P*=0.001), intraluminal thrombosis (*P*=0.02), and macrophage infiltration (*P*=0.05). Plaques from symptomatic patients exhibited a larger necrotic core that occupied >25% of total plaque area, increased angiogenesis, and intraplaque hemorrhage. However, the difference between the groups with regard to those features did not reach statistical significance. Also, the incidences of fibrosis and calcification were not significantly different between groups.

**Figure 1.**
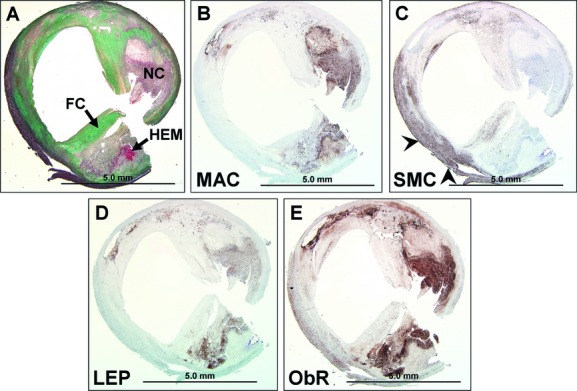
Expression of leptin and its receptor (ObR) in a carotid fibroatheroma complicated by angiogenesis and intraplaque hemorrhage. A, Movat's pentachrome: a fibroatheromatous plaque with a large necrotic core (NC), intraplaque hemorrhage (HEM) (arrow), and overlying thick fibrous cap (FC). B, CD68-positive macrophages (MAC) within and surrounding the necrotic core. C, α-Actin-positive SMCs in the fibrous cap and remnants of media (arrowheads). D and E, Leptin (LEP) and its receptor (ObR), respectively. SMCs indicates smooth muscle cells.

**Figure 2.**
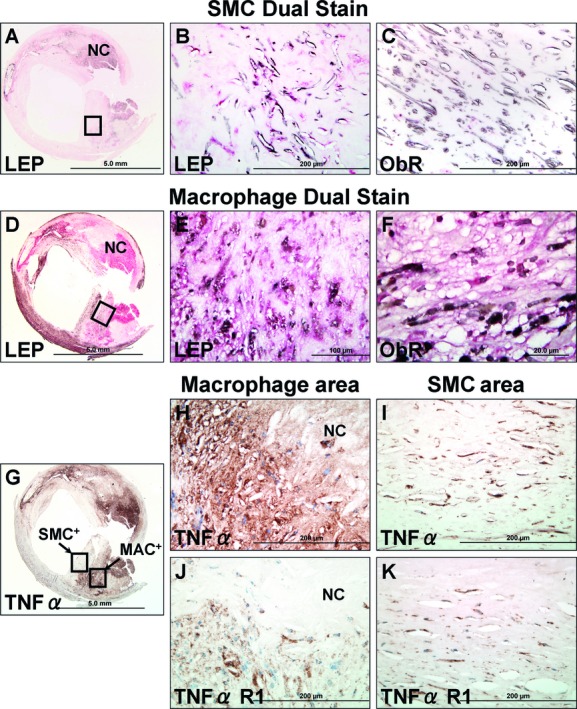
Colocalization of SMCs and macrophages with leptin and its receptor (ObR) and with tumor necrosis factor-α (TNF-α) and its receptor (TNF-αR1) in a human carotid fibroatheroma. A, Dual staining for SMCs (brown-black) and leptin (LEP, red). B, SMCs near the fibrous cap represented by the area within the black box in (A) are positive for leptin. C, SMCs weakly positive for leptin receptors (ObR). D, Dual staining for macrophages (brown-black) and leptin (LEP, red). E and F, Region represented by the area within the black box in (D); macrophages are positive for both leptin and ObR. G, immunostain for TNF-α. H to K, Images within the black boxes in (G) correspond to SMC and macrophage-rich areas (MAC), respectively. TNF-α and its receptor (TNF-αR1) are expressed in both regions. SMCs indicates smooth muscle cells.

The presence of leptin and leptin receptor (ObR) antigen were studied by immunohistochemistry on parallel sections of the carotid plaques. Leptin and ObR were localized to the lesion's shoulder regions and areas within or bordering the necrotic core (Figure 1), also exhibiting colocalization with CD68-positive macrophages. SMCs (as determined by positive α-actin signal) in the fibrous cap also showed staining for leptin and ObR, although this signal was typically less intense. Dual immunostaining verified the cellular colocalization of leptin and ObR primarily in macrophages and SMCs (Figure 2). Further analysis revealed that TNF-α and its receptor TNF-αR1 (isoform 55) were present in similar cell types. Signal from leptin, ObR, TNF-α, and TNF-αR1 was found in plaques from both groups.

**Figure 3.**
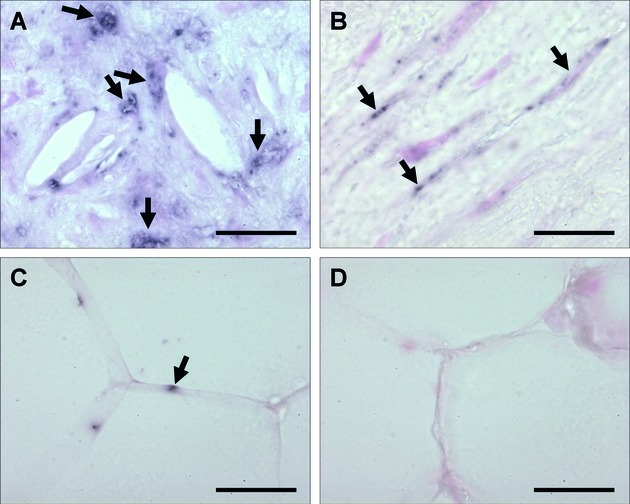
In situ hybridization analysis for leptin mRNA expression in carotid atherosclerotic plaques. A, Human carotid atherosclerotic lesion. Leptin mRNA expression in cells surrounding cholesterol clefts, presumably macrophages. B, Spindle-shaped cells, presumably SMCs, exhibit positive staining for leptin mRNA. C and D, Human adipose tissue, using antisense as positive control (C), or sense probe as negative control (D). Scale bars = 50 μm.

To address the possibility of local leptin synthesis, in situ hybridization analysis was performed and demonstrated the expression of leptin transcript mostly within inflammatory cells, identified as monocytes/macrophages, bordering areas of necrotic core and cholesterol clefts (Figure 3A). In addition, SMCs scattered within the media layer and fibrous cap were also positive for leptin mRNA (Figure 3B).

**Figure 4.**
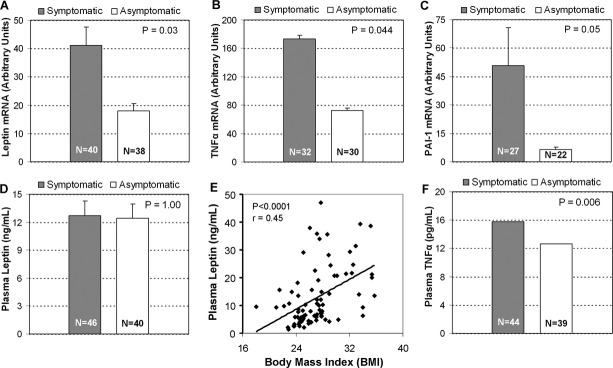
Real-time quantitative PCR analysis of leptin, TNF-α, and PAI-1 mRNA in symptomatic and asymptomatic plaques; plasma levels of leptin and TNF-α. A, Real-time quantitative PCR results for leptin mRNA in both groups were analyzed, revealing a significant difference (*P*=0.03). B, Similar analysis for TNF-α (*P*=0.044). C, Real-time quantitative PCR for PAI-1 mRNA (*P*=0.05). D, Plasma leptin levels were not significantly different in symptomatic and asymptomatic patients (*P*=1.0). E, Scattered plot of plasma leptin level vs body mass index for multiple individual patients of the whole series. The regression curve demonstrates that correlation is statistically significant (*P*<0.0001). F, TNF-α plasma levels were significantly higher in samples from symptomatic vs asymptomatic patients (*P*=0.006). Panels A through D show mean value and standard error.

Leptin mRNA transcript levels in carotid lesions were assessed by quantitative PCR and were found to be significantly increased in symptomatic compared to asymptomatic plaques (41.12 versus 17.95 mean arbitrary units, *P*=0.03; 2-sided Wilcoxon sum test, Figure 4A). Analysis for TNF-α mRNA also yielded a preferential increase in symptomatic lesions (170.60 versus 74.0 mean arbitrary units, *P*=0.044; 2-sided Wilcoxon sum test, Figure 4B). Moreover, mRNA levels of leptin and TNF-α positively correlated in individual plaque samples (*P*=0.004; Pearson's correlation test). Notably, mRNA transcript for PAI-1, the physiological inhibitor of fibrinolysis, was increased in symptomatic plaques (50.6 versus 6.4 mean arbitrary units*, P*=0.05, Fisher exact test, Figure 4C).

Systemic levels of leptin and TNF-α were assessed in plasma samples collected before surgery from neurologically symptomatic and asymptomatic patients. Leptin levels were determined by radioimmunoassay (see Methods) and yielded no difference between symptomatic and asymptomatic groups (12.67±11.04 versus 12.43±9.73 μg/L; *P*=1.0) (Figure 4D). Furthermore, plasma leptin levels correlated with body mass index when assessed per individual sample, for symptomatic and asymptomatic patients (*P*<0.0001; *r*=0.45, Pearson correlation coefficient) (Figure 4E). No correlation was found between leptin levels and arterial hypertension or diabetes mellitus when analyzed for the whole series or separately for each group. Unlike plasma leptin, TNF-α levels assessed by ELISA were significantly higher in samples from symptomatic patients (15.75±7.97 versus 12.67±9.37 pg/mL; *P*=0.006; 2-sided Wilcoxon sum test) (Figure 4F). These results indicate that the presence of leptin in carotid plaques is not a reflection of a systemic induction. Elevated levels of TNF-α within the plaque, however, could be affected by systemic increase.

**Figure 5.**
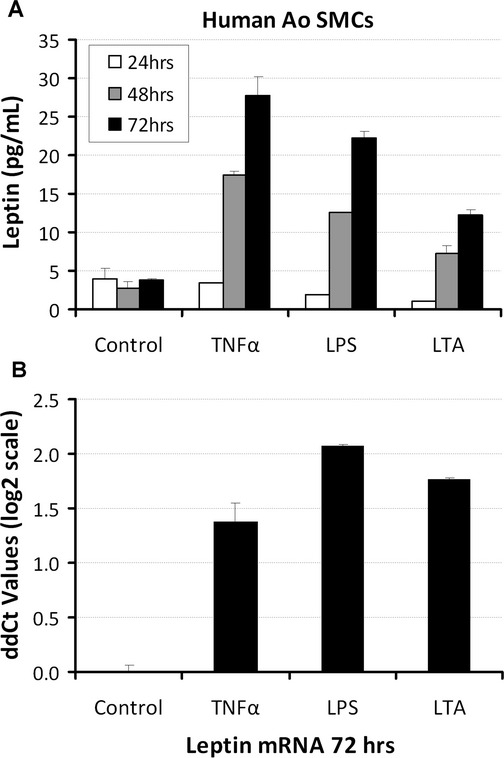
Leptin antigen level in conditioned media from cultured human aortic SMCs upon stimulation by TNF-α, LPS or LTA. A, Representative experiment showing leptin immunoreactivity analyzed in conditioned media of aortic (Ao) SMCs after treatment with 50 ng/mL TNF-α, 100 ng/mL LPS, or 10 μg/mL LTA, determined at 24, 48, and 72 hours. Values were compared to leptin antigen levels in conditioned media from control cultures. B, Quantitative PCR for leptin performed on RNA extracted from human aortic cell lysate of 72 hours samples. For all measurements, values were >5 standard errors higher than the control. Error bars represent standard errors of technical repeats. SMCs indicates smooth muscle cells.

To investigate leptin induction in human vascular SMC cells, both aortic and saphenous vein SMCs were stimulated with TNF-α or bacterial cell wall components (LPS or LTA). Leptin antigen levels were increased in culture media in response to stimulation with any of the 3 agonists. Those were assessed after 24, 48, and 72 hours, exhibiting a similar pattern in both aortic and saphenous vein SMC cultures. There was no increase after 24 hours. Repeated-measures analysis revealed the following fold increases: After treatment with TNF-α (50 ng/mL), there was a 4.93±1.95-fold increase in leptin antigen level after 48 hours (n=3; *P*=0.048) and a 5.95±2.19-fold increase after 72 hours (n=3; *P*=0.042); after treatment with LPS (100 ng/mL), there was a 5.02±2.02-fold increase in leptin antigen level after 48 hours (n=5; *P*=0.005) and a 6.70±2.16-fold increase after 72 hours (n=5; *P*=0.002); and after treatment with LTA (10 μg/mL), there was a 4.2±2.98-fold increase in leptin antigen level after 48 hours (n=5; *P*=0.03) and a 6.04±2.61-fold increase after 72 hours (n=5; *P*=0.006). Results of one representative experiment are displayed in Figure 5A.

At 72 hours, real-time PCR measurements for leptin mRNA showed a clear rise in transcript levels upon treatment with TNF-α, LPS, and LTA, with ddCt values corresponding to >2-, 4-, and 2.5-fold increases, respectively (Figure 5B). The kinetics of transcription at 72 hours correlated with the increased antigen levels in the conditioned media.

## Discussion

This study reveals that leptin mRNA is overexpressed in carotid atherosclerotic plaques from clinically symptomatic patients. Leptin and ObR expression colocalize within CD68-positive macrophages in the shoulder regions bordering the necrotic core and in SMCs of the fibrous cap. Thus, the abundance of de novo synthesized leptin and ObR in macrophages and SMCs within carotid atherosclerotic lesions suggests that locally produced leptin and its receptor facilitate proatherogenic and remodeling processes, contributing to plaque destabilization with clinical events. This assumption also is based on recently collected experimental data from a mouse model of leptin-induced aortic aneurysm, which revealed a direct local degenerative effect on the extracellular matrix attributable to locally applied leptin on the outer aortic wall surface.^36^ Although leptin synthesized in the plaques might have strong local effects, it is unlikely to contribute significantly to the systemic pool of leptin, determined by its synthesis in white adipocytes. In a previous study of carotid plaques from symptomatic and asymptomatic patients, we reported no preferential elevation of plasma leptin nor any increased levels of soluble leptin receptor, C-reactive protein, or interleukin-6 in patients with documented clinical neurological events.^27^ The results given in the present study are in accordance with those previous findings, because leptin activity under specific clinical circumstances might be more decentralized than previously assumed. Thus, in addition to its endocrine activities, leptin could function as a paracrine mediator at the plaque level. Interestingly, an analogous physiological mechanism was demonstrated in a rat portal vein model, in which stretch-dependent local leptin release exerted a direct hypertrophic effect on vascular SMCs.^13^

In the present study, we show that plasma leptin was not significantly different in symptomatic and asymptomatic patients (12.67±11 versus 12.43±9.7 ng/mL, respectively) and was correlated with body mass index. There was no correlation between elevated plasma leptin and documented diabetes mellitus, hypertension, or angiotensin-converting enzyme inhibitor therapy, and the prevalence rates of all these parameters were not significantly different between groups. These findings suggest that the paracrine effects of leptin at the plaque level might not respond to feedback mechanisms related to obesity, insulin resistance, or hypertension.

Interestingly, plaques from both groups were similarly graded according to the AHA classification. However, when Virmani's criteria for plaque vulnerability were applied, a clear tendency toward increased instability was evident in symptomatic lesions. The latter exhibited significantly higher macrophage infiltration, a trend toward higher necrotic core size, and increased incidence of subsequent plaque rupture. To assess additional features characteristic of lesion instability, which also are attributable to symptomatic plaques, we determined PAI-1 mRNA in both groups. PAI-1 is known to correlate with the degree of atherosclerosis.^37^ We found PAI-1 transcript to be significantly increased in the symptomatic plaques in our study, consistent with a previous report.^38^ These results suggest a hypofibrinolytic nature of symptomatic lesions in our series, rendering them unstable and likely to embolize.

Leptin is synthesized primarily by white adipocytes but also is produced in other cell types, such as fibroblasts, placental trophoblasts, skeletal muscle, and gastrointestinal tract epithelium.^39^ We confirmed that carotid plaque macrophages and SMCs are capable of synthesizing leptin. Several modes of leptin induction were demonstrated in adipocytes, including exposure to insulin, dexamethasone,^40^ angiotensin II,^41^ glucose, and hypoxia.^42^ TNF-α and interleukin-1β also were shown to induce leptin in cultured adipocytes and preadipocytes.^43^ Angiotensin II recently was shown to induce leptin in human coronary SMCs^44^ and in human aortic aneurysm wall–derived SMCs in our laboratory.^45^ To examine putative candidate mediators responsible for the local induction of leptin, we chose to assess the presence of TNF-α within both the plaque tissue and the systemic circulation. Our hypothesis about the potential role of TNF-α was based on a range of converging clinical and experimental data implicating this proinflammatory cytokine in atherosclerosis. For instance, plasma TNF-α levels correlate with atherosclerotic disease incidence^46^ and with early carotid atherosclerosis in nonsymptomatic patients.^47^ In addition, chronic exposure to bacterial infections, associated with elevated TNF-α, has been linked to carotid atherosclerosis in a large prospective study.^48^ Analogously, a higher prevalence of recurrent infections was reported in patients suffering from symptomatic coronary atherosclerosis.^49^ Finally, this paradigm is further supported by a study that identified a broad variety of bacterial molecular signatures in coronary atherosclerosis specimens, many of which are known to stimulate TNF-α production.^50^ Our analysis reveals a concomitant preferential increase in TNF-α and leptin mRNA transcripts in carotid plaques from clinically symptomatic patients. Strong signal for TNF-α and its receptor (TNF-αR1) was also evident in carotid plaque macrophages and SMCs. At the systemic level, a concomitant increase in levels of circulating TNF-α antigen were recorded in symptomatic patients. It should be emphasized that plasma samples were collected from all patients 1 to 2 hours before surgery—days to weeks after stroke or transient ischemic attack occurred in symptomatic patients. It is thus unlikely that any elevation of systemic TNF-α level could have resulted from inflammation related to the acute neurological event, plaque rupture, or subsequent instant intraluminal thrombosis. At the same time, this sequence of events could suggest a preceding infection or inflammatory condition that might have promoted both systemic and intraplaque TNF-α synthesis. Notably, elevated levels of TNF-α also could have resulted from activation of resident macrophages by oxidized low-density lipoprotein,^51^ spontaneously released from local lysis of macrophages, or in turn, TNF-α induction by leptin.^52^

The systemic elevation in TNF-α levels in symptomatic patients, especially its fraction within the plaque, could have subsequently induced leptin synthesis locally in a paracrine or autocrine manner. This potential scenario is supported by the abundance of TNF-α and its receptor in macrophages and SMCs and also by the fact that macrophage infiltration was increased in symptomatic plaques. Notably, a previous in vivo study reported leptin production in response to LPS or TNF-α stimulation.^53^ However, this could have resulted from leptin induction in response to stimulation of the adipocyte cell mass. Our cell culture experiments were focused on leptin induction by cytokines in human vascular SMCs, a major cellular component of the vessel wall, and the atherosclerotic plaque. The present in vitro data indicate direct linkage between TNF-α, Gram-negative bacterial LPS, or Gram-positive bacterial LTA and leptin induction in SMCs. Also, the potential presence of a paracrine/autocrine mechanism is suggested from experimental data on the release of TNF-α in response to LPS and TNF-α stimulation in SMC cultures.^54^ Our study does not present leptin induction in primary human macrophage cultures when stimulated with the same agonists or in macrophage cultures exposed to hypoxia (2% oxygen, Hiroyuki Yamasaki, unpublished data). However, it is reasonable to assume that under in vivo conditions, cell-to-cell interaction could provide the essential milieu for leptin induction in macrophages, to account for the presence of leptin mRNA and antigen in carotid plaque macrophages (Figures 2 and 3). Notably, leptin has been shown to facilitate the generation of lipid-laden foam cells via increased formation of cytoplasmic lipid bodies, also increasing the production of inflammatory mediators in macrophages.^55^ Furthermore, an increased number of foam cells within the plaque is likely to augment its vulnerability to rupture.

The linkage between bacterial infection and resulting enhanced atherosclerosis has been classically attributed to the Toll-like receptors (TLRs).^56^ These trans-membrane receptors are involved in the innate immune response system through their unique capability to recognize and interact with highly conserved bacterial, viral, or autoimmune-related pathogen patterns. For example, TLR-4 recognizes LPS, and TLR-2 recognizes LTA. TLRs are expressed by many cell types in atherosclerotic plaques,^57^ and their activation triggers the inflammatory response cascade, promoting the synthesis of proatherogenic cytokines via nuclear factor-κB activation.^58^ Interestingly, leptin might play an essential regulatory role in the expression of TLRs 1 through 9 in preadipocytes and adipocytes.^59^ Thus, leptin could be also induced through TNF-α synthesis *via* the TLR system response.

In summary, leptin and ObR are preferentially synthesized in clinically symptomatic carotid plaques by resident SMCs and macrophages. This induction might be activated by cytokines like TNF-α when available within the plaque and in the systemic circulation. Cytokine upregulation has been speculated to originate from bacterial infection or repeated inflammatory stimulation. Oversynthesized leptin within the plaque could exert paracrine or autocrine effects causing destabilization of the atherosclerotic lesion, resulting in embolic complications.

This study has some limitations. Because plaque tissue was totally consumed for histological analysis and total RNA extraction, none was available for protein extraction. Undoubtedly, quantification of protein levels could have complemented leptin and TNF-α synthesis in situ data. A major disadvantage was the lack of medical records to account for preceding infections or severe inflammation in symptomatic or asymptomatic patients. Moreover, given that leptin might mediate several processes involved in plaque destabilization, the clinical time course from leptin's local synthesis to the occurrence of an embolic event is unclear. Although the present study provides additional data to suggest an alternative avenue to account for the linkages among infection/inflammation, atherosclerosis, and plaque instability, it does not set out to downplay the contribution of other mechanisms involved in plaque destabilization.
